# Factors affecting modern contraceptive use among fecund young women in Bangladesh: does couples’ joint participation in household decision making matter?

**DOI:** 10.1186/s12978-018-0558-8

**Published:** 2018-06-22

**Authors:** Ahmed Zohirul Islam

**Affiliations:** Department of Population Science and Human Resource Development, University of Rajshahi, Rajshahi, 6205 Bangladesh

**Keywords:** Decision making power, women’s empowerment, Young women, Modern contraceptives, Family planning, BDHS

## Abstract

**Objectives:**

The purpose of the study was to explore the association between couples’ joint participation in household decision making and modern contraceptive use (MCU) among fecund (physically able to bear child) young women in Bangladesh.

**Methods:**

This study utilized a cross-sectional data (*n* = 3507) extracted from the Bangladesh Demographic and Health Survey (BDHS) 2011. Differences in the utilization of modern contraceptives (MC) by socio-demographic characteristics were assessed by χ^2^ analyses. Binary logistic regression was used to identify the associated factors of usingMC, and the odds ratio with a 95% CI was computed to assess the strength of association. Multicollinearity was also checked by examining the standard errors in the fitted model.

**Results:**

Desire for a child after two years go by and no child at all contributed the most to increasing MCU followed by receiving family planning (FP) methods from FP workers. Couples’ joint decision making power on women’s health care, child’s health care and visiting family members or relatives emerged as the third most influential factor that might be associated with MCU.

**Conclusions:**

Since spousal joint decision making increases the likelihood of using MC, government should include strategic interventions in FP programs to elevate women’s status through creating educational and employment opportunities and encouraging more visible involvement in household decision making.

## Plain English summary

This study aimed to explore the association between couples’ joint participation in household decision making and modern contraceptive use (MCU) among young women in Bangladesh. Present study utilized a cross-sectional data extracted from the Bangladesh Demographic and Health Survey 2011. A total of 3507 currently married women below 25 years old from total surveyed 17,842 women aged 15–49 years were selected for analyses.

The highest increase of the likelihood of using modern contraceptives (MC) was found among young women who desired a child after two years go by or no child at all, followed by those who were given family planning (FP) methods by FP workers. Remarkably, couples’ joint participation in decision making on women’s health care, child’s health care and visiting family members or relatives emerged as the third most influential factor that might increase the likelihood of usingMC.

In conclusion, government should include strategic interventions in FP programs to lift up women’s status through creating educational and employment opportunities and encouraging more visible involvement in household decision making.

## Introduction

Family planning (FP) is one of the major issues in many developing countries where poor maternal and child health care services are practiced [1, 2] . Studies show that contraceptive use averts 272,040 maternal deaths by reducing the chance of pregnancy and the associated complications (exposure reduction), lowering the risk of having an unsafe abortion (vulnerability reduction), delaying first pregnancy in young women who might have premature pelvic development, and reducing hazards of frailty from high parity and closely spaced pregnancies [3] and prevents almost 230 million births every year worldwide [4]. Statistics showed that an increase of 15 to 17% of using contraceptives reduces population growth by one birth for one woman [5]. However, 62% Bangladeshi women aged 15–49 years use some method of contraception, and 54% use modern methods [6]. Literature shows that women’s lack of power restricts their ability to make decisions about FP practice [7, 8]. Although women’s empowerment is a key to using contraceptives [9], most partners give inferior positions to women in all aspects of decision-making in developing countries [9–11]. Besides, little is known about how participation in household decision-making is associated with the utilization of modern contraceptives (MC) among young women in Bangladesh.

The population of the world became 7336 million in 2015 where South Asia contributed 1834 million people. Bangladesh, the third most populous country in South Asia comprises of 160.4 million people [12], where half of the population is aged below 25 years [13]. As this large cohort of young people enter the reproductive life span, the growth and size of the population of Bangladesh over the next few decades will largely depend on their reproductive behaviour. A considerable number of young adults get married every year. Fulfillment of their contraceptive demand is crucial to the ongoing FP programmes. Therefore, this study focuses on assessing the modern contraceptive use (MCU) status of women under 25 years old.

There is a body of literature that suggests some socio economic and demographic factors, such as residence [14], education [15, 16], age [16], economic status [17], employment status [18], religion [19, 20], parity [21], access to media [15, 19], autonomy [22, 23], desire for children [23], marital status [14] and partner communication [19], have been associated with the use ofMC. In addition, other studies revealed that women’s power and autonomy is favorably related to better reproductive health and use of contraceptives [24–27]. These studies commonly provide evidence for one compelling proposition that women who exhibit substantial autonomy in the household have greater ability to control their body and achieve desired fertility. Decisions about adopting FP such as using contraceptives for either spacing or liming childbirth are often strongly shaped by spousal relationships [28]. Hence, this study attempted to address the question of whether couples’ joint participation in household decision making or women’s independent household decision making is more influential inMCU. Furthermore, since infecund women are physically unable to bear child and pregnant women are not currently in need of using contraceptives, including these women may bias the results. Therefore, this study aimed to address this gap in knowledge by exploring the association between couples’ joint participation in household decision making and use of MC among currently married, fecund (physically able to bear child) and non-pregnant (who were not pregnant during the survey) young women in Bangladesh.

## Methods

### Data sources

This study used a representative set of cross-sectional data extracted from the Bangladesh Demographic and Health Survey (BDHS) 2011. The survey was conducted under the authority of the National Institute of Population Research and Training (NIPORT) of the Ministry of Health and Family Welfare, Bangladesh. Under this survey, Bangladesh was divided into seven administrative regions called divisions such as Barisal, Chittagong, Dhaka, Khulna, Rajshahi, Rangpore and Sylhet. Each division was subdivided into districts, and each district into *upazilas*. Each rural area in an *upazila* was divided into union parishads (UP) and *mouzas* within a UP. An urban area in the *upazila* was divided into *wards*, and into *mohallas* within a ward [29].

According to the official report of BDHS 2011 [29], a total of 47 people were trained to conduct household listing, to delineate Enumeration Areas (EAs), and to administer Community Questionnaires. They were also got training for the use of global positioning system (GPS) units, to obtain locational coordinates for each selected EA. The training hold out for seven days from May 11–21, 2011. A household listing operation was carried out in all selected EAs from May 22 to October 5, 2011 in four phases, each about three weeks in length. Training for the main survey was lasted for four weeks from June 6 to July 5, 2011. A total of 173 fieldworkers were recruited based on their educational level, prior experience with surveys, maturity, and willingness to spend up to six months on the project. Fieldwork for the 2011 BDHS was conducted by 16 interviewing teams, each consisting of one supervisor, one field editor, five female interviewers, two male interviewers, and one logistics staff member. The collection of data was accomplished in five phases, starting on July 8, 2011 and ending on December 27, 2011.

**Fig. 1:**
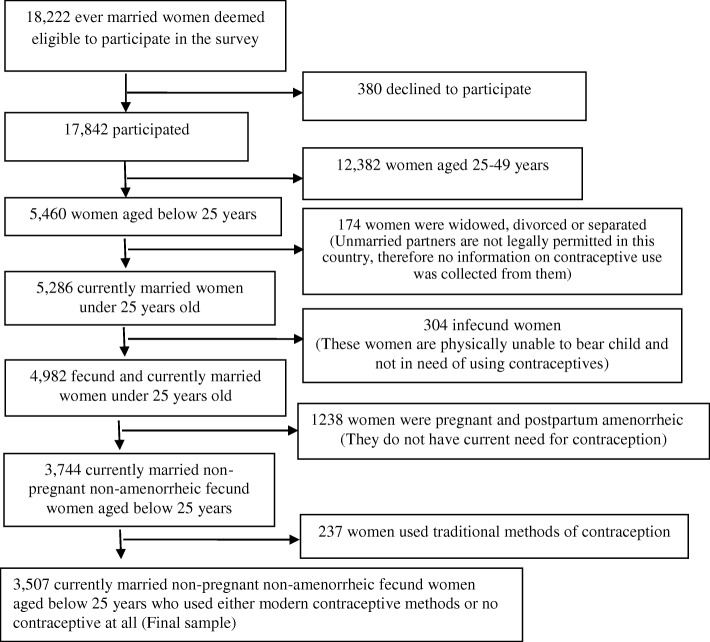
From 17,842 women aged 15–49 years a total of 3507 currently married fecund non-pregnant young women were considered for analyses: BDHS 2011

A nationally representative household based sample was created through a stratified, multistage cluster sampling strategy of which 600 primary sampling units were constructed (207 in urban areas and 393 in rural areas). The primary sampling units were derived from a sampling frame created for the 2011 Population and Housing Census, provided by Bangladesh Bureau of Statistics (BBS). Detailed information on survey design and sampling procedures has been reported elsewhere [29]. A total of 17,842 ever married women aged 15–49 were interviewed in the survey. After applying the definition of young people adopted by the United Nations [30], a total of 3507 currently married non-pregnant fecund women younger than 25 years, who were in actual need of using contraceptives, were selected for analyses (Fig. 1).

### Response variable

The outcome variable of this study wasMCU, which is binary in nature (use or non-use). During the survey, sexually active women were asked if they were currently using any method to delay or avoid getting pregnant. Those that reported using any method to delay or avoid getting pregnant were further asked to indicate what they were doing or the method they were using. MC, such as pills, condoms, Intra-Uterine Devices (IUD), injections and implants/norplants, refer to safe and effective methods to prevent pregnancy [29]. A binary variable is then created and categorized as using any type of MC and versus no use.

### Statistical analyses

This study begins with descriptive exploration of both dependent and independent variables. Differences in MCU by socio-demographic characteristics were assessed by χ^2^ analyses, with significance for all analyses set at *P* <  0.05. Since the response variable of this study had two categories, the binary logistic regression model was fitted to assess the net effect of selected socio-demographic variables. Because stepwise logistic regression analysis is a technique for selecting influential variables in multiple regression models [31], this study used this technique of analysis (backward LR method). All the variables significant in bivariate analyses were simultaneously included in the stepwise logistic regression model, and finally, the most influential predictors for MCU were explored. Multicollinearity in the logistic regression analyses in this study was checked by examining the standard errors for the regression coefficients. A standard error larger than 2.0 indicates numerical problems, such as multicollinearity among the independent variables [32]. All of the independent variables in the fitted model had a standard error <  0.75 that indicate absence of multicollinearity in the study. Missing values are omitted from the analysis. Data were analyzed using SPSS Release 21.0 (SPSS Inc., Chicago, IL).

## Results

### Modern contraceptive use status and socio-demographic profiles

Table 1 describes the percentage distribution of using MC according to socio-demographic characteristics of 3507 currently married women younger than 25 years. Modern contraceptive prevalence was 69% among fecund women aged below 25 years. This study elucidates that 18% of women who wanted a child after two years go by were at risk of having mistimed pregnancies, 15% of women who wanted no child at all were at risk of encountering unwanted pregnancies in their young ages and 47% of women who were undecided about having child were at risk of getting unplanned pregnancies due to not using contraceptives.

**Table 1:** Percentage distribution of modern contraceptive use by socio-demographic characteristics of women: BDHS 2011 (*n* = 3507)

	Modern contraceptive use (%)		*p*-value
Characteristics	No	Yes	
Age (years)			< 0.001
15–19	37.3	62.7	
20–22	28.9	71.1	
23–24	26.6	73.4	
Husband’s age (years)			0.072
15–25	33.7	66.3	
26–30	31.3	68.7	
31–77	29.0	71.0	
Age at marriage (years)			< 0.001
< 15	26.2	73.8	
15–17	31.8	68.2	
18–24	39.6	60.4	
Educational level			0.466
Illiterate	35.4	64.6	
Primary	30.5	69.5	
Secondary	31.1	68.9	
Higher	32.6	67.4	
Religion			0.011
Muslim	32.0	68.0	
Non-Muslim	25.1	74.9	
Place of residence			0.003
Urban	28.2	71.8	
Rural	33.1	66.9	
Region			< 0.001
Barisal	20.9	79.1	
Chittagong	42.8	57.2	
Dhaka	35.2	64.8	
Khulna	25.0	75.0	
Rajshahi	25.2	74.8	
Rangpur	24.3	75.7	
Sylhet	47.7	52.3	
Wealth index			0.109
Poorest	29.1	70.9	
Poorer	28.1	71.9	
Middle	32.8	67.2	
Richer	33.7	66.3	
Richest	32.2	67.8	
Husband’s occupation			< 0.001
Manual	33.8	66.2	
Non-manual	24.5	75.5	
Did not work	38.1	61.9	
Visited by FP worker in past 6 months			< 0.001
Talked	24.3	75.7	
Gave family planning method	4.9	95.1	
Talked and gave method	4.8	95.2	
No	34.1	65.9	
Number of living children			< 0.001
0	61.1	38.9	
1	24.1	75.9	
2	15.5	84.5	
3–5	19.2	80.8	
Desire for more children			< 0.001
Wants within 2 years	76.6	23.4	
Wants after 2+ years	18.0	82.0	
Wants, unsure timing	48.1	51.9	
Undecided	47.0	53.0	
Wants no more	14.9	85.1	
Husband’s desire for children			< 0.001
Both wanted same	30.4	69.6	
Husband wanted more	31.9	68.1	
Husband wanted fewer	26.6	73.4	
Did not know	66.1	33.9	
Person who usually decides on respondent’s health care			< 0.001
Respondent alone	54.0	46.0	
Respondent and husband jointly	22.7	77.3	
Husband alone	28.0	72.0	
Someone else	54.3	45.7	
Other	68.8	31.3	
Person who usually decides on visits to family or relatives			< 0.001
Respondent alone	50.3	49.7	
Respondent and husband jointly	23.4	76.6	
Husband alone	27.9	72.1	
Someone else	49.6	50.4	
Other	57.1	42.9	
Person who usually decides on large household purchases			< 0.001
Respondent alone	52.3	47.7	
Respondent and husband jointly	24.3	75.7	
Husband alone	27.2	72.8	
Someone else	45.9	54.1	
Other	65.0	35.0	
Final say on: Child health care			< 0.001
Respondent alone	45.7	54.3	
Respondent & husband jointly	15.5	84.5	
Husband alone	20.0	80.0	
Someone else	45.8	54.2	
Other	60.8	39.2	
Total	31.4	68.6	

*Note*: Row percentages sum to 100%; *p*-values are based on chi-square tests

This study also revealed several socio-demographic factors that might be associated with MCU at a bivariate level of analysis, using the chi-square test and hence, provided *p*-values for the differences of the groups within each explanatory variable according to the percentages of using versus not using MC (Table 1). MCU was found highest among fecund women aged 23–24 years, those who were non-Muslim, who got married before age of 15 years, who had two or more living children, those whose husbands were professional non-manual workers, who lived in Barisal division, who resided in the urban areas and who jointly took decision with their husbands regarding their own health care, child health care, large household purchases and visiting to family members or relatives.

### Factors affecting modern contraceptive use of young women

Stepwise logistic regression analyses were performed to identify the most influential factors that might affect the likelihood of using MC. Table 2 shows that the highest increase of the likelihood of using MC was found among young women who desired a child after two years go by or no child at all, followed by those who were given FP methods by FP workers, who took decision together with their husbands about women’s own healthcare, child’s healthcare, and visiting family members or relatives, who had increasing number of living children, whose husbands were professional workers, and who were non-Muslim. This study also indicated that the greatest decrease of the likelihood of using MC was observed among women who lived in Sylhet or Chittagong region, followed by women who did not know about their husbands’ desires for the number of children and who lived in the rural areas of the country.

**Table 2:** Logistic regression model for modern contraceptive use

Predictors	Odds Ratio (95% CI)	*p*-value
Person who usually decides on respondent’s health care		
Respondent alone ®		< 0.001
Respondent and husband jointly	2.761 (1.925–3.960)	< 0.001
Husband alone	2.698 (1.875–3.885)	< 0.001
Someone else	1.186 (0.770–1.827)	0.439
Other	2.445 (0.577–10.368)	0.225
Final say on: Child health care		
Respondent alone ®		< 0.001
Respondent & husband jointly	2.616 (1.808–3.786)	< 0.001
Husband alone	2.164 (1.430–3.277)	< 0.001
Someone else	1.156 (0.739–1.808)	0.526
Other	1.524 (0.982–2.364)	0.060
Person who usually decides on visiting family or relatives		
Respondent alone ®		0.065
Respondent and husband jointly	1.830 (1.176–2.849)	0.007
Husband alone	1.559 (0.992–2.449)	0.054
Someone else	1.377 (0.851–2.229)	0.193
Other	0.876 (0.255–3.012)	0.833
Desire for more children		
Wants within 2 years ®		< 0.001
Wants after 2+ years	14.004 (10.841–18.091)	< 0.001
Wants, unsure timing	5.949 (3.032–11.674)	< 0.001
Undecided	3.477 (1.902–6.358)	< 0.001
Wants no more	10.854 (7.517–15.674)	< 0.001
Visited by FP worker in past 6 months		
Talked ®		< 0.001
Gave family planning method	5.047 (2.151–11.840)	< 0.001
Talked and gave method	4.479 (1.228–16.335)	0.023
No	0.781 (0.546–1.117)	0.176
Husband’s occupation		
Manual ®		< 0.001
Non-manual	1.701 (1.354–2.138)	< 0.001
Did not work	1.264 (0.711–2.248)	0.425
Husband’s desire for children		
Both want same ®		0.010
Husband wants more	0.761 (0.522–1.108)	0.154
Husband wants fewer	1.080 (0.734–1.589)	0.697
Did not know	0.462 (0.281–0.760)	0.002
Religion		
Muslim®		
Non-Muslim	1.628 (1.134–2.336)	0.008
Place of residence		
Urban ®		
Rural	0.617 (0.502–0.759)	< 0.001
Region		
Barisal ®		< 0.001
Chittagong	0.259 (0.179–0.375)	< 0.001
Dhaka	0.479 (0.333–0.690)	< 0.001
Khulna	0.790 (0.536–1.166)	0.235
Rajshahi	0.871 (0.585–1.298)	0.498
Rangpur	0.589 (0.396–0.877)	0.009
Sylhet	0.231 (0.150–0.358)	< 0.001
Age	0.932 (0.883–0.984)	0.011
Age at marriage	1.077 (1.018–1.140)	0.010
Number of living children	1.951 (1.516–2.511)	< 0.001
Constant	0.105	< 0.001

*Note*: ® Reference category, CI is confidence interval

We observed that women who desired a child after two years go by were more likely to use MC [OR(95% CI): 14.004 (10.841–18.091), *p* <  0.001] and who wanted no child at all were also more likely to use MC [OR(95% CI): 10.854 (7.517–15.674), *p* <  0.001] than who desired a child within two years go by. Besides, women who were undecided about having a child were 3.477 times and who wanted another child but unsure about timing were 5.949 times likely to use MC compared to those who wanted another child within two years go by. Women who were given FP methods by the FP workers were 5.047 [(95% CI): (2.151–11.840), *p* = < 0.001] times likely to use MC than their counterparts who only talked with the FP workers.

The likelihood of using MC was increased when husband and wife jointly participate in decision making on respondent’s own health care [OR (95% CI): 2.761 (1.925–3.960), *p* <  0.001], on child health care [OR (95% CI): 2.616 (1.808–3.786), p <  0.001], and on visiting family members or relatives [OR (95% CI): 1.830 (1.176–2.849), *p* = 0.007] than their counterparts who decided alone about these issues. It was also observed that if decisions about health care of women, child health care and visiting family members or relatives were taken by husband alone then the likelihood of using MC was increased by 2.698 times, 2.164 times and 1.559 times respectively compared to the women who took decision alone about these matters.

We also observed that number of living children played a significant role in contraceptive use since the likelihood of using MC was increased [OR (95% CI): 1.951 (1.516–2.511), *p* <  0.001] with increasing the number of children. The likelihood of using MC increased [OR (95% CI): 1.701 (1.354–2.138), *p* <  0.001] among women whose husbands were professional (non-manual) workers compared to their counterparts whose husbands were manual workers. Non-Muslim women were more likely to use MC than their Muslim counterparts.

Women residing in Sylhet, Chittagong, Dhaka, and Rangpur division were 0.231, 0.259, 0.479, and 0.589 times likely to use modern contraceptives than who lived in Barisal division, respectively. Women who resided in the rural areas were less likely to use [OR (95% CI): 0.617 (0.502–0.759), *p* <  0.001] modern contraceptives than who lived in the urban areas. The likelihood of using modern contraceptives was decreased among women who did not know about their husbands’ desires for having children than those women who desired equal number of children with their husbands’ desire. The likelihood of using MC was decreased with increasing age and increased with increasing age at marriage.

## Discussion

This study assessed the relationship between household decision making and MCU and also identified the other factors contributed to the utilization of MC among currently married, fecund and non-pregnant women under 25 years old in Bangladesh using Demographic and Health Survey data of 2011.

Results showed that consensus in household decision making regarding women’s own health care, visiting family members or relatives and child health care appeared as the third, fourth and sixth most influential contributing factor of MCU, respectively. The most significant factors contributed to using modern contraceptives were desire for children after two years, or want no child at all and receiving FP methods from FP workers. Other key factors that showed significant variability in using MC were number of living children, husbands engaged in professional non-manual jobs, regional variations and place of residence.

Present study demonstrated that women who were under collective decision-making with their husbands regarding their own health care, child health care and visiting family members or relatives were more likely to use MC than those who took decision alone about these matters. The likelihood of using MC decreased among young women who did not know about their husbands’ desires for having children in comparison to women who shared the same feelings regarding parenthood as their husbands. Hence, this study clearly indicated that communication between husband and wife and eventually couples’ joint participation in household decision making emerged as one of the most influential factors that might be associated with MCU. Studies suggest that greater gender equality may encourage women’s autonomy and may facilitate the uptake of contraception because of increased female participation in decision making [33]. Moreover, male participation in sharing the responsibility to practice and support family planning is identified as a vital strategy in increasing the contraceptive prevalence rate [34].

This study also revealed that women who desired another child after two years go by were more likely to use MC than those women who desired another child within two years go by. This finding is similar to previous study conducted in Bangladesh, Uganda and Pakistan [35–37]. Some of the reasons for this postponed childbearing as stated in other studies are: women’s increased participation on the labour market, including their longer education [38–40] and career planning [38]. Furthermore, financial and practical circumstances during their studies may be difficult to combine with establishing a family, and a high educational level and a desire for career development and will increase the likelihood of delaying child birth in women [40–42]. Young women often express a need to avoid pregnancy because they may be too young to care for a baby, they may have to end or postpone their education [43].

Women who were given family planning methods by the FP workers were more likely to use MC than their counterparts who only talked with the FP workers. Consistently, other studies done in Bangladesh and Cambodia highlighted that outreach activities by FP workers and accessibility to FP related information to married women of reproductive age were significantly associated with use of modern contraceptives [19, 44, 45]. The likelihood of using MC was increased with increasing number of children. This finding is in line with the previous reports from Bangladesh, Tanzania and Pakistan [36, 37, 46].

This study described that women in Sylhet Chittagong, and Dhaka divisions were less likely to use MC than women living in Barisal division. Residing in the rural areas decreased the likelihood of using MC in comparison to their urban counterparts. Studies suggest that geographical variations in the utilization of contraceptives have been found to be influenced by a number of factors like cultural beliefs such as, value attached to child [47], the presence and quality of reproductive health care services [48], the physical characteristics of the area, and the presence of transport routes [49, 50].

Women whose husbands did professional jobs were more likely to use MC compared to women whose husbands were manual workers. This study supports findings from several other studies that showed that Muslim women were less likely to use MC than their non-Muslim counterparts [19, 37]. The likelihood of using MC was decreased with increasing age and increased with increasing age at marriage among fecund women aged below 25 years. Therefore, establishment of youth-friendly service centers in convenient places and providing essential materials would encourage young people to use reproductive health services [45].

### Limitations

This study must be considered with some limitations. There may be possibility of threats to internal validity. Firstly, major portions of the observations were dropped (approximately 79%) during data cleaning because this study considered only currently married, non-pregnant and fecund young women. Therefore, this study would suffer from the selection bias. Secondly, the possibility of under reporting cannot be ruled out since young women may be reluctant to reveal their contraceptive use status. Because, though the government and non-government organizations have a long history of investment in FP, contraceptive use is still a sensitive and often stigmatized subject in Bangladesh [37]. However, the personal interview method applied in this study is widely resorted to for this kind of research.

There may also be a possibility of threat to external validity. Although nationally representative data set was employed, this study cannot be generalized to all women in Bangladesh because it focused on only currently married and fecund young women. Finally, the questionnaire was filled out by the interviewers and their personal opinions might have biased the information. However, according to the BDHS report, interviewers were provided training for implementing the survey based on a training manual especially developed to enable the field staff to collect data in a friendly, secure, and ethical manner. In spite of these limitations, this study revealed important associations between couples’ consensus in decision making and MCU, which have significant implications. Nevertheless, since a nationally representative dataset is used in this study, findings could be a true representation of the situation of this sub-group of women. Moreover, international comparisons of results are possible as DHS surveys take up similar instruments across the countries.

## Conclusions

This study concludes that spousal joint participation in household decision making emerged to be a significant factor contributed to increasing the likelihood of MCU. Therefore, policy makers should focus on developing negotiation skills in young people by creating educational and employment opportunities. Government should include strategic interventions in FP programs to elevate women’s status through encouraging more visible involvement in household decision making in order to increase MCU. Besides, desire for a child after two years go by or no child at all contributed the most to increasing the likelihood of MCU, followed by getting FP methods from FP workers. Therefore, this study also suggests that FP interventions should be tailored through wide spreading the activities of FP workers and introducing reproductive and sex education in schools to prepare the young for healthy and responsible living. Because remarkable portions of fecund young women want to either postpone or delay pregnancies but do not use contraceptives, this study suggests a qualitative study to investigate in depth why this sub-group of women does not use contraceptives. In last but not least, contraceptive discontinuation and switching of methods among young and older women would be our future research.

## Abbreviations

BDHS: Bangladesh Demographic and Health Survey
CI: Confidence Interval
FP: Family planning
MC: Modern contraceptives
MCU: Modern contraceptive use
OR: Odds Ratio
